# Bildgebende Diagnostik bei Erkrankungen des Dünndarms

**DOI:** 10.1007/s00117-026-01592-9

**Published:** 2026-03-12

**Authors:** Martina Scharitzer, Thomas Mang, Nino Bogveradze, Ulrike Attenberger

**Affiliations:** Universitätsklinik für Radiologie und Nuklearmedizin, Medizinische Universität Wien, Währinger Gürtel 18–20, 1090 Wien, Österreich

**Keywords:** Schnittbildgebung, Intestinale Neoplasien, Entzündliche Darmerkrankungen, Mesenteriale Ischämie, Darmobstruktion, Cross-sectional imaging, Intestinal neoplasms, Inflammatory bowel diseases, Mesenteric ischemia, Intestinal obstruction

## Abstract

Die Diagnostik von Dünndarmerkrankungen stellt eine besondere Herausforderung dar, da dieser Darmabschnitt endoskopisch nur eingeschränkt zugänglich ist und die klinische Symptomatik häufig unspezifisch bleibt. Zudem wird die Beurteilung durch die Darmmotilität sowie die Abhängigkeit von einer Kontrastmittelgabe und einer luminalen Distension zusätzlich erschwert. Moderne Schnittbildverfahren, insbesondere die CT(Computertomographie)- und MR(Magnetresonanz)-Enterographie, haben die bildgebende Diagnostik von entzündlichen, neoplastischen und vaskulären Dünndarmerkrankungen revolutioniert. Die präzise Differenzierung der verschiedenen Pathologien des Dünndarms erfordert fundierte Kenntnisse charakteristischer Bildgebungsmuster. Wandverdickungen, deren Ausdehnung, Verteilung und Kontrastmittelaufnahmemuster, mesenteriale Gefäßveränderungen und begleitende extraintestinale Befunde liefern entscheidende diagnostische Hinweise. Die Integration klinischer Parameter mit morphologischen und funktionellen Bildgebungskriterien ermöglicht eine zeitnahe und zielgerichtete Therapieplanung.

## Abstract (en)

The diagnostics of small bowel diseases represent a special challenge as this intestinal segment is not readily accessible endoscopically and the clinical symptoms are often nonspecific. The assessment is further complicated by the bowel motility and the dependency on contrast agent administration and luminal distension. Modern cross-sectional imaging techniques, particularly computed tomography (CT) and magnetic resonance (MR) enterography, have revolutionized the diagnostics of inflammatory, neoplastic and vascular small bowel diseases. The precise differentiation of the various small bowel pathologies requires thorough knowledge of characteristic imaging patterns. Bowel wall thickening, the extent, distribution and pattern of contrast enhancement uptake, mesenteric vascular changes and associated extraintestinal findings provide crucial diagnostic indications. The integration of clinical parameters with morphological and functional imaging criteria enables timely and targeted treatment planning.

## Lernziele

Dieser Übersichtsartikel fasst die differenzialdiagnostischen Bildgebungsmerkmale der wichtigsten Dünndarmerkrankungen zusammen und bietet evidenzbasierte Entscheidungshilfen für die radiologische Routine. Nach Absolvieren dieser Fortbildungseinheit …

- können Sie entzündliche Darmerkrankungen erkennen und ihre Aktivität anhand bildgebender Kriterien einschätzen
- sind Sie in der Lage, in akuten klinischen Situationen wie bei gastrointestinaler Blutung, mechanischer Obstruktion und mesenterialer Ischämie therapieentscheidende radiologische Informationen zu liefern.
- kennen Sie die differenzialdiagnostischen Bildgebungsmerkmale benigner und maligner Dünndarmtumoren.
- verstehen Sie die Integration klinischer und bildgebender Parameter zur präzisen Differenzierung der wichtigsten Dünndarmerkrankungen im radiologischen Alltag.

## Einleitung

Die bildgebende Diagnostik des Gastrointestinaltrakts hat durch moderne CT(**Computertomographie**

Computertomographie

)-Geräte und Weiterentwicklungen der abdominalen **Magnetresonanztomographie**

Magnetresonanztomographie

(MRT) einen Paradigmenwechsel erfahren. Die Wahl der optimalen Modalität richtet sich nach der klinischen Situation, möglichen Differenzialdiagnosen und Patientenfaktoren. Nachfolgend werden verschiedene Dünndarmerkrankungen in Bezug auf ihre charakteristischen bildgebenden Merkmale unter Integration klinischer Informationen und ihrer Bedeutung für die weitere individualisierte Therapieplanung diskutiert.

### Fallbeispiel

**Abb. 1:**
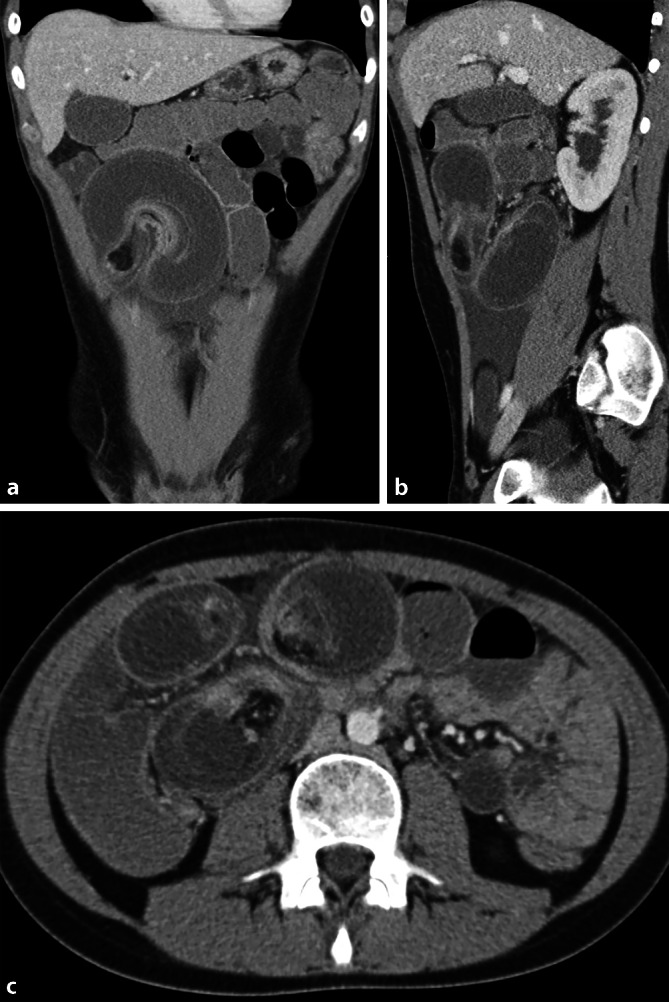
Venöse Computertomographie in koronaler (**a**), sagittaler (**b**) und axialer (**c**) Rekonstruktion

Ein 16-jähriger Patient kommt in die Notfallaufnahme mit seit 2 Tagen zunehmenden Bauchschmerzen und Erbrechen. Neben erhöhten Entzündungswerten (C-reaktives Protein [CRP]: 9 mg/dl, Normwert: < 1 mg/dl) sind die übrigen Laborwerte unauffällig. Aufgrund des klinischen Bildes eines akuten Abdomens wird eine CT in der venösen Phase durchgeführt (Abb. 1).

## Tumoren

Obwohl der Dünndarm mehr als 90 % der mukosalen Oberfläche des Gastrointestinaltrakts ausmacht, entfallen nur weniger als 5 % der gastrointestinalen Tumoren auf dieses Organ [1]. Die Inzidenz dieser Tumoren nimmt zu, während die Mortalitätsrate weitgehend unverändert bleibt [1]. Aufgrund der eingeschränkten endoskopischen Zugänglichkeit und der unspezifischen klinischen Symptomatik kommt der Radiologie eine zentrale Rolle bei Detektion, Charakterisierung, Staging und Verlaufskontrolle zu [2].

Dünndarmtumoren treten meist solitär auf, können jedoch im Rahmen hereditärer Syndrome wie beispielsweise einer **familiären adenomatösen Polypose**

Familiäre adenomatöse Polypose

oder eines **Peutz-Jeghers-Syndroms**

**Abb. 2:**
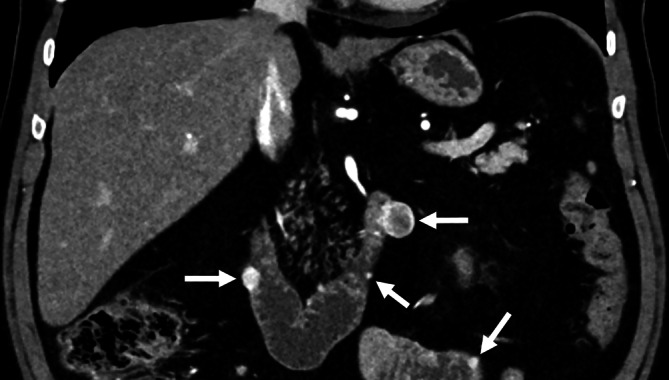
53-jähriger Patient mit Neurofibromatose Typ I ohne abdominale Symptome. Die Computertomographie (CT) in der arteriellen Phase zeigt mehrere hypervaskularisierte, scharf begrenzte, exophytische Läsionen von Duodenum und Jejunum (*Pfeile*), assoziierten GIST(gastrointestinale Stromatumoren)-Tumoren entsprechend

Peutz-Jeghers-Syndrom

auch multipel vorkommen (Abb. 2). Die meisten Läsionen bleiben über lange Zeit klinisch asymptomatisch und werden oft erst zufällig im Rahmen operativer Eingriffe oder bildgebender Untersuchungen entdeckt.

Die topographische Verteilung der verschiedenen Tumorentitäten im Dünndarm zeigt charakteristische Muster (Tab. 1). Mögliche Komplikationen sind die **Dünndarmobstruktion**

Dünndarmobstruktion

und die **enteroenterale Invagination**

Enteroenterale Invagination.

Letztere wird mehrheitlich durch benigne Raumforderungen wie Polypen, Lipome, Leiomyome oder Meckel-Divertikel verursacht [3]. In der abdominalen CT können charakteristische Zeichen wie das **Target-**

Target-Zeichen

oder das **Doughnut-Zeichen**

Doughnut-Zeichen

mit konzentrischen Wandschichten unterschiedlicher Dichte bzw. das **Bowel-within-a-bowel-Zeichen**

Bowel-within-a-bowel-Zeichen

auf eine Dünndarmintussuszeption hinweisen. Aufgrund einer Passagebehinderung kann es zu einer **prästenotischen Dünndarmdilatation**

Prästenotische Dünndarmdilatation

kommen.

**Tab. 1:** Typische Merkmale von Dünndarmtumoren

	Häufige Lokalisation	Typische Merkmale	KM-Verhalten	Assoziierte Befunde	
*Adenom*	Jejunum	Intraluminal, mukosal, weichteildicht	Kräftig homogen	–	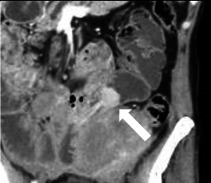
*Lipom*	Jejunum + Ileum	Fetthaltig, dünner mukosaler Überzug	Nicht relevant	–	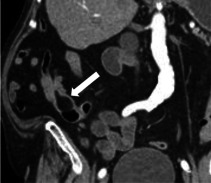
*Adenokarzinom*	Duodenum > Jejunum > Ileum	Zirkumferent, kurzstreckig, Schulterbildung	Moderat heterogen	Regionale LK-SBL	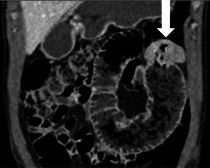
*GIST*	Jejunum	Exophytisch, weichteildicht, gut begrenzt	Heterogen	Keine LK-SBL, hypervaskularisierte Leber-SBL	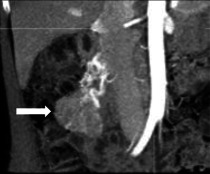
*Lymphom*	Jejunum + Ileum	Deutliche Wanddicke, geringe KM-Aufnahme, aneurysmatische Dilatation, keine Obstruktion	Homogen	Splenomegalie, mesenteriale LK	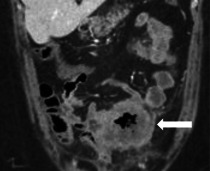
*NET*	Distales Ileum	Kleines Primum, mesenteriale LK-SBL + desmoplastische Reaktion, Verkalkung, Kinking	Kräftig arteriell	Karzinoidsyndrom, Leber-SBL	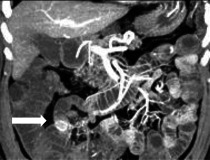

*LK* Lymphknoten, *SBL* sekundär-blastomatöse Läsionen, *GIST* gastrointestinale Stromatumoren, *KM* Kontrastmittel, *NET* neuroendokrine Tumoren

**Abb. 3:**
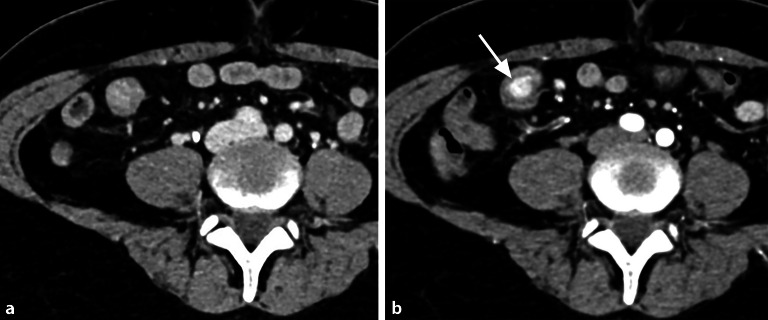
Intraluminaler Tumor des Dünndarms (neuroendokriner Tumor, NET), der in der venösen Computertomographie (CT; **a**) nicht eindeutig, in der arteriellen Phase (**b**) jedoch als hypervaskularisierte intraluminale Läsion deutlich abgrenzbar ist (*Pfeil*)

Manche Tumoren wie neuroendokrine Tumoren (NET) und gastrointestinale Stromatumoren (GIST) weisen eine signifikante arterielle Hypervaskularisation auf, die eine Untersuchung in der arteriellen Phase erfordert (Abb. 3).

### Merke

Transiente Invaginationen können bei Untersuchungen mit starker Füllung und erhöhter Peristaltik (Enterographieprotokoll) v. a. bei Patient:innen mit Zöliakie oder M. Crohn beobachtet werden. Sie unterscheiden sich von pathologischen Invaginationen durch eine kürzere Länge, ein fehlendes Hypomochlion sowie fehlende begleitende Pathologien der Darmwand und deren Umgebung [4].

### Adenokarzinome

Adenokarzinome des Dünndarms sind selten und finden sich am häufigsten im Duodenum. Sie können durch ein zirkumferentes konzentrisches Wachstum rasch zu einer Dünndarmobstruktion führen. Charakteristisch ist eine irreguläre Wandverdickung mit weichteildichter Struktur, inhomogenem Enhancement und einer sog. Schulterbildung, die den abrupten Übergang des Tumors zur normalen Darmwand beschreibt. Begleitend finden sich häufig eine prästenotische Dilatation, mesenteriale Lymphknoten und bei fortgeschrittenen Stadien Fernmetastasen in Leber und Peritoneum.

### Neuroendokrine Tumoren

NET, früher **Karzinoide**

**Abb. 4:**
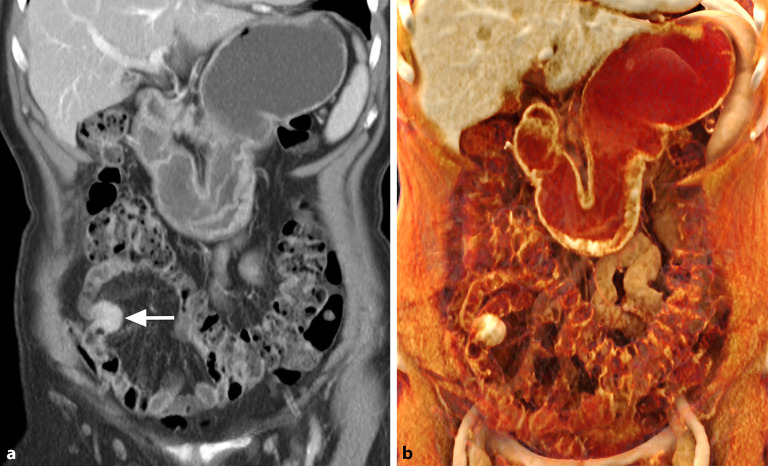
72-jährige Patientin mit unspezifischen abdominalen Symptomen: Die koronale venöse CT(Computertomographie)-Abbildung (**a**) zeigt einen hypervaskularisierten Tumor des Dünndarms (*Pfeil*) mit charakteristischer Verziehung („Haarnadelzeichen“); **b** 3-dimensionale Rekonstruktion dieses Tumors

Karzinoide

genannt, sind häufig im Ileum lokalisiert. Sie werden anhand der Tumorzellproliferation und des Ki67(Kiel 67)-Index in gut differenzierte G1- bis G3-Tumoren und schlecht differenzierte neuroendokrine Karzinome klassifiziert [5]. Der Primärtumor ist meist unscheinbar und klein, weist aber ein starkes arterielles Enhancement auf. Die fibrotische Komponente des Tumors kann zu einer Retraktion der Dünndarmschlinge führen mit typischer „Haarnadelformation“ (Abb. 4). Charakteristisch ist die oft wesentlich ausgeprägtere lymphknotenmetastasenassoziierte **desmoplastische Reaktion**

desmoplastische Reaktion

des Mesenteriums, die zentrale Verkalkungen aufweisen kann [6].

Die Darstellung von **Somatostatinrezeptoren**

Somatostatinrezeptoren

mittels spezifischer Radiopharmaka (Somatostatinanaloga DOTATATE, DOTATOC und DOTANOC) in der Hybridbildgebung ist diagnostisch wegweisend für SSTR(Somatostatinrezeptor)-affine G1- oder G2-Tumoren und Voraussetzung für eine nachfolgende **Radiopeptidrezeptortherapie**

Radiopeptidrezeptortherapie

[7]. Eine FDG(Fluordesoxyglukose)/PET(Positronenemissionstomographie)-CT ist bei neuroendokrinen G3-Karzinomen für das initiale Staging, das Restaging und den Rezidivnachweis indiziert [8]. Der publizierte NETPET-Score ermöglicht durch eine Kombination der SSTR-Expression und der FDG-Avidität eine verbesserte Tumorcharakterisierung mittels Dual-tracer-Untersuchung [9].

### Gastrointestinale Stromatumoren

Diese Tumorgruppe zeigt ein heterogenes radiologisches Erscheinungsbild mit variablem Kontrastmittelverhalten, endoluminalem oder exophytischem Wachstumsmuster und fehlender Umgebungsreaktion. Meist präsentieren sich GIST als gut abgrenzbare Läsionen mit häufig zentral hypodensen Arealen, die Nekrosen oder zystische Umwandlungen repräsentieren [10]. Gastrointestinale Blutungen stellen häufig die Erstmanifestation dar. Für die Beurteilung des Therapieansprechens ist neben der Größenbestimmung die Evaluation der Tumorvitalität entscheidend. Gemäß **Choi-Kriterien**

Choi-Kriterien

entspricht eine Dichteabnahme in der CT von mehr als 15 % oder eine Größenreduktion von mehr als 10 % einem Ansprechen [11]. Bei Lebermetastasen ist nach erfolgreicher Chemotherapie und konsekutiv nekrotischer Umwandlung auf das intraläsionale Auftreten eines Nodule-within-a-mass-Phänomens zu achten, das auf ein Tumorrezidiv hinweisen kann [12].

### Lymphome

Das primäre gastrointestinale Lymphom stellt die häufigste extranodale Manifestation eines **Non-Hodgkin-Lymphoms vom B‑Zell-Typ**

Non-Hodgkin-Lymphom vom B‑Zell-Typ

dar. Radiologisch zeigt sich typischerweise eine homogene, oftmals sehr ausgeprägte konzentrische Wandverdickung mit vermindertem oder fehlendem Kontrastmittel-Enhancement (**„graue Darmwand“**

„Graue Darmwand“;

[13]). Durch die tumoröse Infiltration des myenterischen Plexus und der Muskularis entsteht häufig eine **pseudoaneurysmatische Wachstumsform**

Pseudoaneurysmatische Wachstumsform,

die trotz erheblicher Tumormasse selten zu einer intestinalen Obstruktion führt [14]. **Enteropathieassoziierte T‑Zell-Lymphome**

Enteropathieassoziierte T‑Zell-Lymphome

(EATL) treten bevorzugt bei Zöliakiepatient:innen auf. Neben der Wachstumsform von Lymphomen sind die begleitende Splenomegalie und mesenteriale Lymphknotenkonglomerate charakteristische Befunde.

### Metastasen

**Abb. 5:**
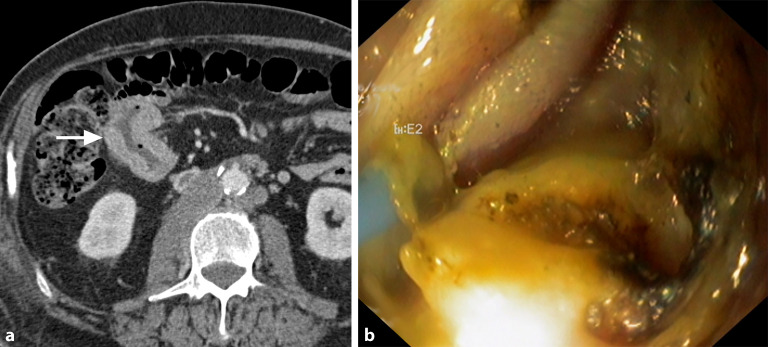
76-jähriger Patient: Als Zufallsbefund bei einer Staging-Untersuchung bei bekanntem Melanom zeigt sich in der venösen Computertomographie eine kurzstreckige Raumforderung des Ileums (*Pfeil*) (**a**), die endoskopisch (**b**) als Melanommetastase verifiziert wurde. (Bildquelle: Innere Medizin III, Medizinische Universität Wien)

Den häufigsten Primärtumor für Dünndarmmetastasen stellt das Melanom dar, gefolgt von Bronchialkarzinomen und Nierenzellkarzinomen. In der CT zeigen sich Metastasen meist als multiple, glatt begrenzte Läsionen, wobei das Enhancement-Muster vom Primärtumor abhängig ist (Abb. 5). Bei einer Peritonealkarzinose lagern sich die Tumorformationen im Gegensatz zu hämatogenen Metastasen als serosale Auflagerungen von außen an die Dünndarmschlingen an und zeigen häufig ein noduläres, girlandenförmiges Verteilungsmuster entlang der mesenterialen Anheftung. Bei Patient:innen mit einem bekannten Malignom ist bei Diagnostik einer Dünndarmraumforderung primär an das Vorliegen einer sekundär-blastomatösen Läsion zu denken.

## Entzündungen

### Infektiöse Dünndarmerkrankungen

Das radiologische Erscheinungsbild infektiöser Dünndarmerkrankungen ist typischerweise uncharakteristisch mit ödematöser Wandverdickung, mukosalem Enhancement und mesenterialer Lymphadenopathie und erlaubt keine spezifische ätiologische Zuordnung. Bestimmte Erreger (*Yersinia, Mycobacterium tuberculosis, Salmonella*) weisen jedoch eine Prädilektion für das terminale Ileum auf, was bei der Differenzialdiagnose ileozökaler Pathologien relevant ist.

### Autoimmune Darmerkrankungen

Autoimmune Erkrankungen des Gastrointestinaltrakts zeigen eine zunehmende Prävalenz [15]. Die häufigsten primären Autoimmunerkrankungen des Dünndarms sind **chronisch-entzündliche Darmerkrankungen**

**Abb. 6:**
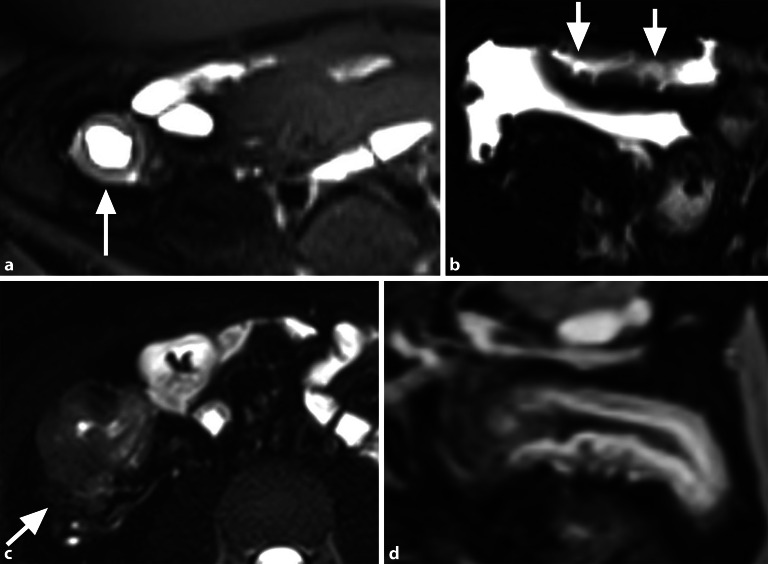
Zeichen einer aktiven chronisch-entzündlichen Darmerkrankung: **a** murales Ödem als T2-hyperintense Signalintensität der Darmwand (*Pfeil*); **b** lineare Ulzera des neoterminalen Ileums (*Pfeile*); **c** T2-hyperintenses Ödem um das terminale Ileum (*Pfeil*); **d** deutlich erhöhte Kontrastmittelaufnahme mit Schießscheibenzeichen des terminalen Ileums

**Abb. 7:**
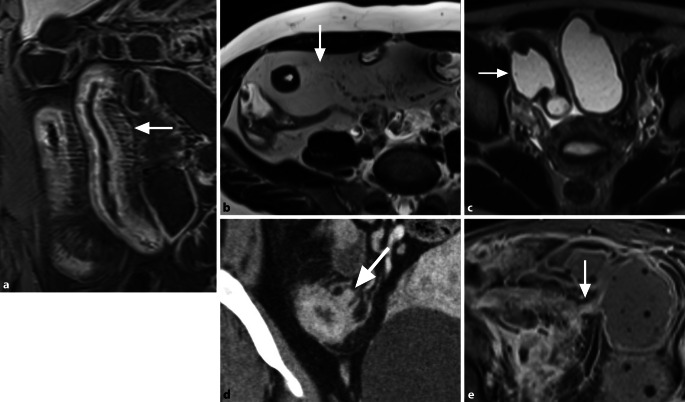
Weitere Entzündungszeichen einer chronisch-entzündlichen Darmerkrankung: **a** „comb sign“ (*Pfeil*); **b** „creeping fat“ (*Pfeil*) um das terminale Ileum; **c** antimesenteriale Sakkulation (*Pfeil*) mit entzündlicher Wandverdickung der dem Mesenterium zugewandten Seite; **d** Sinustrakt (*Pfeil*); **e** enteroenterale Fistel (*Pfeil*)

Chronisch-entzündliche Darmerkrankungen

(CED). Es werden 3 Phänotypen unterschieden: inflammatorisch, stenosierend und fistulierend. Die Differenzierung zwischen aktiv-entzündlichen und chronisch-fibrotischen Wandveränderungen ist therapeutisch entscheidend, da aktiv-entzündliche Läsionen medikamentös behandelbar sind, fibrotische Stenosen jedoch eine chirurgische Resektion erfordern. Da beide Komponenten simultan auftreten und ineinander übergehen können, sollte eine Beurteilung des prädominanten Phänotyps erfolgen. Tab. 2 definiert Zeichen der aktiven Entzündung (Abb. 6) und unspezifische Entzündungszeichen (Abb. 7), die unabhängig von der Krankheitsaktivität sowohl in akuten als auch in chronischen Phasen nachweisbar sind.

**Tab. 2:** Charakteristische Zeichen entzündlicher Veränderungen des Dünndarms

Pathologie	Definition
**Zeichen einer aktiven Entzündung**	
*Murales Ödem*	Intramural ↑ T2-Signal (Fatsat-Sequenz) vgl. mit Muskulatur; kann Schichtungsphänomen verursachen
*Perienterales Ödem*	↑ T2-Signal um die Darmschlinge
*Ulzera*	Fokaler Defekt der Mukosa mit intramuraler Ausbreitung luminaler Inhalte
*Darmwandverdickung*	> 3 mm Wanddicke
*Hyper-vaskularisation*	Verstärkte KM-Aufnahme der Darmwand (oft „Target-Zeichen“)
**Weitere Entzündungszeichen**	
*„Comb sign“*	Vermehrte + erweiterte Vasa recta auf Post-KM-Sequenzen
*„Creeping fat“/„fat wrapping“*	Chronische Hypertrophie des mesenterialen Fettgewebes; Mesenteric Creeping Fat Index (Skala 1–8; [16])
*Antimesenteriale Sakkulation*	Pouch-artige Ausstülpung antimesenterial durch chronische Entzündung und fibrotische Schrumpfung der mesenterialen Seite
*Stenose*	Trias aus > 25 % Wandverdickung, fixierter luminaler Stenose > 50 % und prästenotischer Dilatation (> 3 cm); gemäß SAR-Kriterien prästenotische Dilatation ab > 2,5 cm
*Sinustrakt*	Beginnend fistulierende, die Darmwand überschreitende penetrierende Veränderung, ohne Kontakt zu anderer epithelialisierter Oberfläche [17]
*Fistel*	Simple Fistel: einfache enteroenterale/enterokolische Verbindung Komplexe Fistel: sternförmiges System oder Verbindung zum Urogenitaltrakt
*Motilität*	Reduzierte Peristaltik als Folge einer Entzündung

*fatsat* fettgesättigt, *KM* Kontrastmittel, *SAR* Society of Abdominal Radiology

Der **Magnetic Resonance Index of Activity**

Magnetic Resonance Index of Activity

(MaRIA bzw. „simplified MaRIA“) als häufigster in klinischen Studien angewandter Score quantifiziert die Entzündungsaktivität [18], wird jedoch in der klinischen Routine wenig verwendet. Das **Therapieansprechen**

Therapieansprechen

wird gemäß ECCO(European Crohnʼs and Colitis Organisation)-ESGAR(European Society of Gastrointestinal and Abdominal Radiology)-Empfehlungen als transmurale Remission, signifikantes transmurales Ansprechen, stabile oder progressive Erkrankung klassifiziert [19].

Weitere primär autoimmune Dünndarmerkrankungen umfassen die **Zöliakie**

**Abb. 8:**
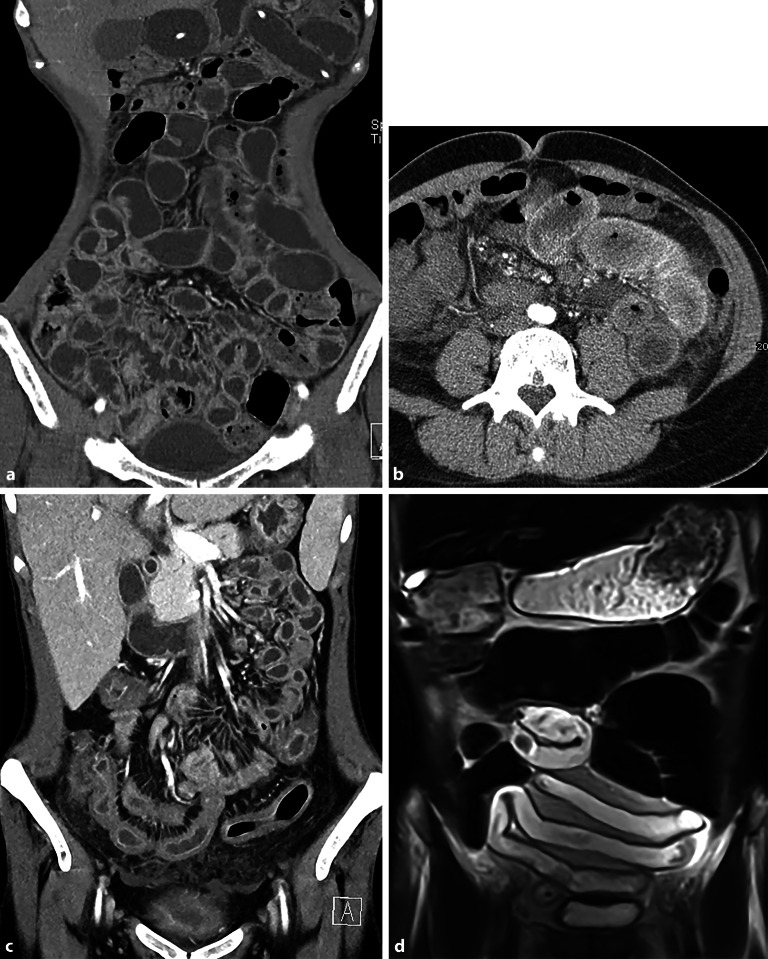
**a** CT(Computertomographie)-Enteroklysma zeigt eine Zöliakie mit Faltenverlust jejunal und Faltenvermehrung ileal; **b** CT arteriell axial: serosale Form einer eosinophilen Enteritis mit verstärkt serosalem Kontrastmittel-Enhancement und mesenterialem Ödem; **c** CT venös koronal: akute GvHD („graft-versus-host disease“) mit flüssigkeitsmarkierten Dünndarmschlingen und deutlich hypervaskularisierter Darmwandverdickung; **d** MR(Magnetresonanz)-Enterographie mit dem chronischen Bild einer GvHD mit Faltenverlust und bandförmig angeordneten Dünndarmschlingen

Zöliakie

mit charakteristischer Inversion des jejunoilealen Faltenreliefs (Abb. 8a), die **eosinophile Enteritis**

Eosinophile Enteritis

in mukosaler, muskulärer oder serosaler Manifestation (Abb. 8b) – typischerweise assoziiert mit einer allergischen Anamnese und einer Eosinophilie – sowie die seltene **IgG4(Immunglobulin G4)-assoziierte Enterokolitis**

IgG4(Immunglobulin G4)-assoziierte Enterokolitis

mit einem unspezifischen radiologischen Erscheinungsbild.

**Sekundäre autoimmune Dünndarmerkrankungen**

Sekundäre autoimmune Dünndarmerkrankungen

treten bei Kollagenosen wie Morbus Behçet oder systemischen Vaskulitiden auf. Die akute intestinale **„graft-versus-host disease“**

„Graft-versus-host disease“

(GvHD) manifestiert sich 10 bis 40 Tage nach Stammzelltransplantation mit flüssigkeitsgefüllten Dünndarmschlingen, Wandverdickung und verstärktem mukosalen Enhancement bei meist fehlender mesenterialer Lymphadenopathie (Abb. 8c). Die späte Form der GvHD (> 100 Tage) zeigt eine Wandverdickung mit multifokalen Strikturen [20]. Der Faltenverlust führt zum charakteristischen „ribbon sign“, einem Erscheinungsbild eines faltenfreien bandförmigen Dünndarms (Abb. 8d).

Differenzialdiagnostisch müssen bei Patient:innen nach **Stammzelltransplantation**

Stammzelltransplantation

verschiedene Pathologien berücksichtigt werden, die sich jedoch in klinischer Anamnese und Lokalisationsmustern von der GvHD unterscheiden. Abzugrenzen ist besonders die CMV(Zytomegalievirus)-Kolitis. Diese betrifft jedoch nur das Kolon und zeigt im Gegensatz zur GvHD bei deutlicher Wandverdickung keine flüssigkeitsgefüllten Darmschlingen. Weitere Alternativdiagnosen schließen die medikamenteninduzierte Enterokolitis, virale Enteritiden, bakterielle Infektionen und die neutropenische Kolitis ein, die sich bevorzugt im Zökum manifestiert und zeitgleich mit der Neutropenie korreliert.

### Andere

Die **Divertikulose**

Divertikulose

des Dünndarms weist mit einer Inzidenz von 0,3–2,3 % eine geringe Prävalenz auf und wird überwiegend bei Komplikationen wie Divertikulitis, Blutungen, intestinaler Obstruktion oder Perforation symptomatisch [21]. **Pseudodivertikel**

Pseudodivertikel,

bestehend aus Mukosa und Submukosa ohne Beteiligung der Muskularis, werden als Ausstülpungen der Darmwand meist als Zufallsbefund in der CT diagnostiziert. Diese sind typischerweise mesenterialseitig und bevorzugt im oberen Dünndarm lokalisiert.

Das **Meckel-Divertikel**

Meckel-Divertikel

stellt als echtes Divertikel eine embryologische Persistenz des Ductus omphaloentericus dar. Die klinische Relevanz lässt sich durch das „Gesetz der 2“ beschreiben: Es betrifft etwa 2 % der Bevölkerung, tritt im Verhältnis 2:1 (männlich zu weiblich) auf, ist typischerweise 2 Inch (ca. 5 cm) lang und manifestiert sich häufig in den ersten beiden Lebensjahren. Eine ^99m^Technetium-Pertechnetat-Szintigraphie ermöglicht den Nachweis ektoper Magenschleimhaut im Meckel-Divertikel.

## Ischämie und Vaskulitis

Mesenteriale Ischämien entstehen in bis zu 50 % durch arterielle Embolien, in 20–30 % durch arterielle Thrombosen und seltener durch venöse Thrombosen oder nichtobstruktive mesenteriale Ischämien (NOMI). Letztere sind mit einer höheren Mortalität assoziiert [22]. Aufgrund der rasch progredienten Darmnekrose mit einem Mortalitätsrisiko von über 80 % nach mehr als 24 h ist eine zeitgerechte radiologische Diagnostik entscheidend [23].

Im **Frühstadium der Darmischämie**

Frühstadium der Darmischämie

zeigt sich intraoperativ eine reversible Schädigung der Mukosa mit einem spastischen Darm und blassen Mesenterium, gefolgt von einer Schädigung der Muskularis und Hypotonie mit Wandausdünnung. Das Spätstadium ist durch eine transmurale Nekrose mit Pneumatose und dem klinischen Bild einer Paralyse und Schocksymptomatik charakterisiert [24].

Pathophysiologisch unterscheiden sich **NOMI**

NOMI

und **obstruktive mesenteriale Ischämie**

(Nicht‑)Obstruktive mesenteriale Ischämie (N)OMI

(OMI) fundamental: Bei der NOMI besteht initial ein Low-flow-Status mit Gefäßspasmen, während bei der OMI diese Minderperfusion inflammationsbedingt im Spätstadium auftritt. Die **Pneumatose**

Pneumatose

entwickelt sich bei einer NOMI früher und kann zu einer Überschätzung des Nekroseausmaßes führen, wohingegen sie bei der OMI ein Spätstadium signalisiert [25].

Typische **radiologische Ischämiezeichen**

Radiologische Ischämiezeichen

umfassen ein reduziertes oder gänzlich fehlendes Wand-Enhancement, eine Dilatation mit Wandausdünnung und die bereits erwähnte Pneumatosis intestinalis. Die Dual-energy-CT kann mittels Jod-Maps die Detektion minderperfundierter Areale erleichtern [26]. Bei der Beurteilung der Kontrastmittelaufnahme ist die physiologisch unterschiedliche Jodkonzentration in verschiedenen Darmabschnitten zu berücksichtigen, abnehmend von Magen/Duodenum zu Rektum [27].

### Merke

Bei gleichzeitigem Vorhandensein unterschiedlicher Schweregrade einer intestinalen Ischämie in verschiedenen Darmabschnitten eines Patienten sind die korrekte Identifikation und Einschätzung des am schwersten betroffenen Areals entscheidend, da dies das weitere Management bestimmt.

## Obstruktion

Der Großteil von Dünndarmobstruktionen wird durch Briden verursacht, gefolgt von inneren Hernien, Tumoren und anderen Ursachen. Bei bariatrischen Patient:innen ist jedoch die innere Hernie mit 42 % die führende Ursache einer Dünndarmobstruktion [28]. Bei häufig relativ unspezifischen klinischen Symptomen in dieser Patient:innengruppe [29] ist bei inneren Hernien an die meist fehlende pathologische Distension der betroffenen Darmschlingen zu denken [30]. Man unterscheidet **Single-loop-**

Single-loop-Obstruktionen

von **Closed-loop-Obstruktionen**

Closed-loop-Obstruktionen.

Während Single-loop-Obstruktionen durch eine singuläre Obstruktionsstelle charakterisiert sind, entsteht eine Closed-loop-Obstruktion (ca. 19 % aller Dünndarmobstruktionen) durch Blockade an 2 Darmabschnitten mit Ausbildung einer dazwischenliegenden geschlossenen Schleife. Bei Letzterer besteht ein erhöhtes Ischämierisiko durch externe Kompression des mesenterialen Gefäßstiels, Distension des „closed loop“ und möglichem Volvulus mit mesenterialer Torsion [31].

**Typische CT-Zeichen**

Typische CT-Zeichen

einer Single-loop-Obstruktion umfassen das „beak sign“ (vogelschnabelartige Zuspitzung am Kalibersprung) und das „fat notch sign“ (Einsenkung von mesenterialem Fettgewebe am Übergangsbereich). Closed-loop-Obstruktionen zeigen durch 2 Obstruktionsstellen häufig eine C‑förmige Konfiguration der betroffenen Darmschlinge und eine radiale Ausrichtung der mesenterialen Gefäße zu den Obstruktionspunkten.

**Abb. 9:**
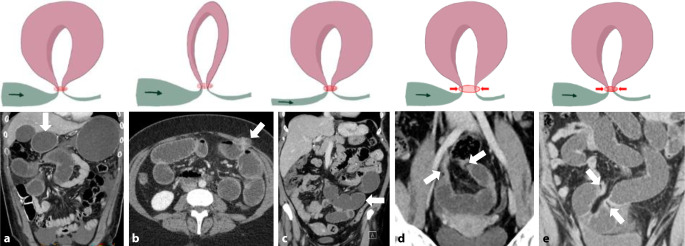
Closed-loop-Obstruktionen [31]: **a** erweiterte zuführende Darmschlinge (*Pfeil*); **b** kollabierte Closed-loop-Schlinge (*Pfeil*) mit prästenotischer Dilatation; **c** innere Hernie (*Pfeil*) mit fehlender prästenotischer Dilatation (Flat-belly-Typ); **d** weiter Abstand (*Pfeile*) zwischen den beiden Obstruktionszonen; **e** Abstand von < 8 mm (*Pfeile*) zwischen den Transitionszonen

Spezifische Befunde bei Closed-loop-Obstruktionen umfassen eine bereits nativ hyperdense Darmwand als Hinweis auf eine irreversible transmurale Nekrose und einen hyperdensen Darminhalt mit HU(Hounsfield Units)-Werten von mehr als 11 im Vergleich zu nichtbetroffenen Darmschlingen [32]. In 70 % zeigt sich eine Dilatation der zuführenden Schlinge, in 25 % ein Kollaps der betroffenen Darmschlinge und in weniger als 5 % keine prästenotische Dilatation der zuführenden Schlinge (Abb. 9a–c; [31]). Ein Abstand von weniger als 8 mm zwischen den Transitionszonen ist mit einer erhöhten Notwendigkeit einer Operation beschrieben worden (Abb. 9d, e; [31]).

### Merke

Eine lokalisierte Darmparalyse kann eine beginnende Dünndarmobstruktion imitieren. Meist wird sie durch entzündliche Veränderungen benachbarter Organe verursacht (z. B. Pankreatitis mit erweiterten Dünndarmschlingen im linken Oberbauch). Diese betroffenen aperistaltischen Dünndarmschlingen werden auch als Sentinel-loop-Schlingen bezeichnet.

## Blutung

Die **CT-Angiographie in der arteriellen Phase**

CT-Angiographie in der arteriellen Phase

hat sich als die sensitivste Methode für die Detektion einer gastrointestinalen Blutung erwiesen [33]. Voraussetzung für die Detektion eines intraluminalen Kontrastmittelextravasats ist eine Blutungsrate von zumindest 0,1 ml/min [34]. Durch die Dual-energy-CT-Technik kann eine virtuelle Non-contrast-Serie die echte native Sequenz ersetzen. Die jodbasierte Karte (Jod-Mapping) kann die Genauigkeit in der Blutungsdetektion erhöhen und die Wahrscheinlichkeit einer korrekten Identifikation des Blutungsorts erhöhen [35]. Ein Areal erhöhter Dichte auf der nativen Sequenz (45–70 HU) wird als **„sentinel clot sign“**

„Sentinel clot sign“

bezeichnet und weist durch den höheren Hämatokritgehalt des akut geronnenen Bluts auf die anatomische Ursprungsstelle der aktiven Blutung hin [36].

### Merke

Intraluminale hyperdense Darminhalte (Nahrungsmittelreste, Medikamente mit Bismuth- oder Kalziumgehalt, orales Kontrastmittel) oder Nahtmaterial können Kontrastmittelextravasate simulieren und damit akute Blutungen vortäuschen. Im Vergleich zu Blutungen sind diese bereits auf nativen Bildern sichtbar. Daher ist der Vergleich von CT-Bildern vor und nach Kontrastmittelgabe zu empfehlen.

## Auflösung Kasuistik

Die CT zeigt das typische Erscheinungsbild einer **Dünndarminvagination**

Dünndarminvagination.

Neben invaginierten mesenterialen Gefäßen konnte eine umschriebene fetthaltige Läsion an der Spitze der Invagination identifiziert werden (mesenteriales Fettgewebe). Intraoperativ bestätigte sich die Verdachtsdiagnose eines Volvulus, ausgelöst durch ein invaginiertes **Meckel-Divertikel**

**Abb. 10:**
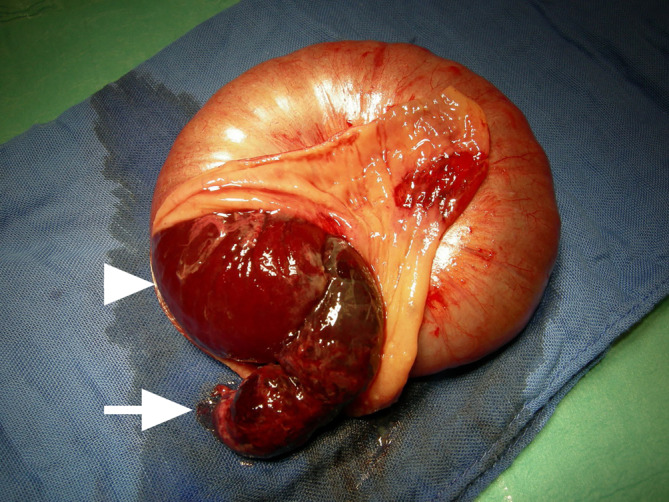
Intraoperativer Befund: Bestätigung eines Dünndarmvolvulus mit ursächlich invaginiertem Meckel-Divertikel (*Pfeil*) mit hämorrhagischer Alteration des invaginierten Darmsegments (*Pfeilspitze*). (Bildquelle: Kinderchirurgie, Medizinische Universität Wien)

Meckel-Divertikel

(Abb. 10). Das betroffene Dünndarmsegment zeigte sich massiv aufgetrieben und hämorrhagisch alteriert, sodass eine Resektion erforderlich war. Differenzialdiagnostisch ist an ein ileales Lipom als alternative Ursache der Invagination zu denken.

## Fazit für die Praxis

- Die bildgebende Diagnostik ermöglicht eine differenzierte Beurteilung des gesamten Spektrums von Dünndarmerkrankungen – von entzündlichen Prozessen über benigne und maligne Tumoren bis hin zu vaskulären und obstruktiven Pathologien.
- Die Beurteilung charakteristischer Merkmale wie Wandverdickung, Kontrastmittelaufnahmemuster, Wachstumsform und mesenteriale Veränderungen erlaubt eine gezielte Eingrenzung möglicher Differenzialdiagnosen intestinaler Tumoren.
- Bei akuten Erkrankungen wie Ischämie, Obstruktion und Blutung liefert die Computertomographiebildgebung managemententscheidende Informationen für interventionelle, konservative oder chirurgische Therapieoptionen.

## CME-Fragebogen

Der Dünndarm macht mehr als 90 % der mukosalen Oberfläche des GI-Traktes aus. Wie hoch ist der Anteil an gastrointestestinalen Tumoren im Dünndarm? Weniger als...

1 %

3 %

5 %

10 %

15 %

Welches bildgebende Merkmal ist charakteristisch für neuroendokrine Tumoren des Dünndarms?

Zirkumferentes konzentrisches Wachstum mit rascher Obstruktion

Pseudoaneurysmatische Wachstumsform ohne Obstruktion

Desmoplastische mesenteriale Reaktion mit möglichen Verkalkungen

Exophytisches, gut begrenztes Wachstum ohne Umgebungsreaktion

Fetthaltige intraluminale Raumforderung

Welche Tracer-Kombination wird beim NETPET-Score zur verbesserten Charakterisierung neuroendokriner Tumoren (NET) verwendet?

PSMA (prostataspezifisches Membranantigen) und FDG (Fluordesoxyglukose)

SSTR(Somatostatinrezeptor)-Tracer und FDG

Cholin und PSMA

DOTATOC und Cholin

FDG und Gallium-68

Welche Kriterien sind für die Beurteilung des Therapieansprechens bei gastrointestinalen Stromatumoren nach Chemotherapie in der Computertomographie am geeignetsten?

Größenreduktion um mindestens 30 % nach RECIST (Response Evaluation Criteria in Solid Tumors)

Größenreduktion > 10 % oder Dichteabnahme > 15 %

Vollständige Kontrastmittelaufnahme des Tumors

Fehlen von Lebermetastasen im Follow-up

Normalisierung der Tumormarker

Welcher Befund in der Computertomographie ist ein spezifisches Zeichen für eine irreversible transmurale Nekrose bei einer Closed-loop-Obstruktion?

Prästenotische Dilatation

„Beak sign“ am Kalibersprung

Bereits nativ hyperdense Darmwand

C‑förmige Konfiguration der Darmschlinge

Fat-notch-Zeichen

Welches der folgenden radiologischen Merkmale ist *kein* spezifisches Zeichen einer aktiven Entzündung bei Morbus Crohn?

Ulkus

Murales Ödem

„Creeping fat“

Perienterales Ödem

Verstärkte Vaskularisation

Bei einem Patienten nach bariatrischer Operation mit klinischem Verdacht auf Dünndarmobstruktion ist welche Ursache am wahrscheinlichsten?

Adhäsionen mit Bridenileus

Innere Hernie

Dünndarmtumor

Intussuszeption

Meckel-Divertikel

Eine 48-jährige Patientin entwickelt 6 Monate nach allogener Stammzelltransplantation rezidivierende abdominale Schmerzen und Diarrhö. Die Magnetresonanzenterographie zeigt eine Dünndarmwandverdickung mit multiplen kurzen Stenosen und Verlust der normalen Schleimhautfaltung. Diese Befunde sind am ehesten vereinbar mit:

Akute intestinale GvHD („graft-versus-host disease“)

Chronisch intestinale GvHD

Morbus Crohn

CMV(Zytomegalievirus)-Enteritis

Intestinales Lymphom

Ein 72-jähriger Patient auf der Intensivstation unter Katecholamintherapie entwickelt abdominale Schmerzen. Die CT(Computertomographie)-Angiographie wird zum Ausschluss einer mesenterialen Ischämie durchgeführt. Welches vaskuläre Erscheinungsbild ist charakteristisch für eine nichtobstruktive mesenteriale Ischämie (NOMI)?

Atherosklerotische Plaques mit hochgradiger Stenose der A. mesenterica superior

Intravaskulärer Embolus im Hauptstamm der A. mesenterica superior

Isolierte Dissektion der A. mesenterica superior

Kollateralkreisläufe als Zeichen chronischer Insuffizienz der A. mesenterica superior

Diffuse Spasmen der A. mesenterica superior

Welche Entitäten zählen *nicht* zu den primär autoimmunen Darmerkrankungen?

Graft-versus-Host-Disease

Chronisch entzündliche Darmerkrankungen

Zöliakie

Eosinophile Enteritis

IgG4-assoziierte Enterokolitis
